# Recent Advances in the Therapeutic and Diagnostic Use of Liposomes and Carbon Nanomaterials in Ischemic Stroke

**DOI:** 10.3389/fnins.2018.00453

**Published:** 2018-07-05

**Authors:** Lorena F. Fernandes, Gisele E. Bruch, André R. Massensini, Frédéric Frézard

**Affiliations:** Departamento de Fisiologia e Biofísica, Instituto de Ciências Biológicas, Universidade Federal de Minas Gerais, Belo Horizonte, Brazil

**Keywords:** nanocarrier, liposomes, carbon nanotubes, graphene, fullerenes, stroke, nanobiosensor, imaging

## Abstract

The complexity of the central nervous system (CNS), its limited self-repairing capacity and the ineffective delivery of most CNS drugs to the brain contribute to the irreversible and progressive nature of many neurological diseases and also the severity of the outcome. Therefore, neurological disorders belong to the group of pathologies with the greatest need of new technologies for diagnostics and therapeutics. In this scenario, nanotechnology has emerged with innovative and promising biomaterials and tools. This review focuses on ischemic stroke, being one of the major causes of death and serious long-term disabilities worldwide, and the recent advances in the study of liposomes and carbon nanomaterials for therapeutic and diagnostic purposes. Ischemic stroke occurs when blood flow to the brain is insufficient to meet metabolic demand, leading to a cascade of physiopathological events in the CNS including local blood brain barrier (BBB) disruption. However, to date, the only treatment approved by the FDA for this pathology is based on the potentially toxic tissue plasminogen activator. The techniques currently available for diagnosis of stroke also lack sensitivity. Liposomes and carbon nanomaterials were selected for comparison in this review, because of their very distinct characteristics and ranges of applications. Liposomes represent a biomimetic system, with composition, structural organization and properties very similar to biological membranes. On the other hand, carbon nanomaterials, which are not naturally encountered in the human body, exhibit new modes of interaction with biological molecules and systems, resulting in unique pharmacological properties. In the last years, several neuroprotective agents have been evaluated under the encapsulated form in liposomes, in experimental models of stroke. Effective drug delivery to the brain and neuroprotection were achieved using stealth liposomes bearing targeting ligands onto their surface for brain endothelial cells and ischemic tissues receptors. Carbon nanomaterials including nanotubes, fullerenes and graphene, started to be investigated and potential applications for therapy, biosensing and imaging have been identified based on their antioxidant action, their intrinsic photoluminescence, their ability to cross the BBB, transitorily decrease the BBB paracellular tightness, carry oligonucleotides and cells and induce cell differentiation. The potential future developments in the field are finally discussed.

## Introduction

Stroke is one of the leading causes of death and disability worldwide. According to the most recent meetings of the American Heart Association the global prevalence of stroke was 42.4 million in 2015. Ischemic stroke was 24.9 million, and hemorrhagic stroke was 18.7 million in the entire world. Stroke incidence in the United States each year shows that approximately 610,000 people experience a first stroke attack and 185,000 are recurrent attacks (87% are ischemic and 10% are intracerebral hemorrhage strokes, whereas 3% are subarachnoid hemorrhage strokes cases). Direct and indirect costs with stroke accounted 40.1 billions of dollars between 2013 and 2014 (Benjamin et al., 2018).

Stroke is defined by a sudden decrease of blood supply to the brain tissue. It can result in the manifestation of symptoms like numbness of face, arms and legs, confusion, aphasia, among others. Signs and symptoms will depend on the affected area and can have an outcome ranging from complete patient recovery to severe neurological deficits and death (Feuerstein and Wang, 2000; Casals et al., 2011; Kyle and Saha, 2014). It can be divided in two different categories: ischemic and hemorrhagic. Ischemic stroke or cerebral ischemia is characterized by disruption of blood flow to the brain as a result of an obstruction in the arteries that irrigate a certain area of the brain. This obstruction can be caused by a clot or a plunger (Luo et al., 2017). The hemorrhagic stroke is less common and divided into intracranial, when a rupture of an artery causes extravasation of blood to the cerebral parenchyma (Mayer and Rincon, 2005); or subarachnoid, in which the blood leakage is usually caused by some trauma or ruptured aneurysm (Rafii and Hillis, 2006).

In this review, we will focus on ischemic stroke and discuss the current diagnostic and therapeutic challenges. Despite the great incidence, the severity of the outcomes and the high economic costs of ischemic stroke, tissue plasminogen activator (t-PA) is the only treatment approved by the Food and Drug Administration (FDA). However, due to safety concerns such as the risk of cerebral hemorrhage after treatment with t-PA, the number of patients who can actually use this drug is very low (Adeoye et al., 2011). In addition, even when blood flow is restored, secondary damage caused by reperfusion can be observed in brain tissue, mainly because of the production of deleterious substances, such as reactive oxygen species (ROS) and inflammatory cytokines (Iadecola and Anrather, 2011). In the case of ischemic stroke and also many other central nervous system (CNS) disorders, the current treatment and development of new therapies have been limited by the following factors: (1) ineffective delivery of most CNS drugs into the brain parenchyma because of the blood brain barrier (BBB), hindering preventive treatment or the rescue of areas not yet totally affected (Alyautdin et al., 2014); (2) poor stability or toxicity of a large number of drugs after systemic and/or oral administration; (3) insufficient understanding of the physiopathology of the disease; (4) difficulty to translate good results from pre-clinical studies to the clinic.

In this scenario, nanotechnology emerges with innovative tools for therapeutic, diagnostic and theranostic purposes. These tools include, for instance, imaging techniques, implants, sensors, biomarkers, drug development and carrier systems and biomaterials (Hong et al., 2015). Regarding treatment and diagnosis of ischemic stroke, nanomaterials can act in at least three different forms: (1) as a drug carrier, avoiding drug degradation and unspecific binding to sites where it may exert toxicity and facilitating its passage across the BBB (Fukuta et al., 2016); (2) through its specific characteristics and resulting pharmacological actions, for instance as an antioxidant (Lee et al., 2011); (3) as a diagnostic tool or building blocks in the development of sensors for several biomolecules (from ROS to neurotransmitters) (Kruss et al., 2013).

After a brief description of the physiopathology and the current limitations of the diagnosis and therapy of ischemic stroke, this review highlights the recent progress achieved in the use of two important types of nanostructures: liposomes as first generation of drug nanocarriers, but still the most advanced and studied; carbon nanomaterials recently investigated as neuroprotective agents, membrane-permeable and permeabilizing agents, cell and drug-carrier systems and innovative biosensors for mechanistic evaluation in experimental models. These two types of nanostructures were selected for comparison in this review because of their very distinct characteristics and ranges of applications. Liposomes represent a biomimetic system, with composition, structural organization and properties very similar to biological membranes. On the other hand, carbon nanomaterials, which are not naturally encountered in the human body, exhibit new modes of interaction with biological molecules and systems, resulting in unique pharmacological properties.

## Physiopathology of Ischemic Stroke

After cerebral ischemia, there is an interruption of blood supply to the brain, which induces a shortage of oxygen and glucose delivery, compromising the production of ATP by oxidative phosphorylation (Deb et al., 2010). A shift toward anaerobic glycolysis also occurs which results in the lowering of brain tissue pH (Anderson et al., 1999; Liu et al., 2011). As neurons lack energy store, they are vulnerable to this reduction in oxygen and glucose (Krol et al., 2013). The reduction in ATP levels causes energy imbalance and consequently the cells have difficulty to maintain ionic homeostasis. Membrane potential is lost and neurons and glia depolarize increasing the levels of Na^+^ and Ca^2+^ entering the cell (Katsura et al., 1994). This depolarization leads to a release of excitatory amino acids to the extracellular space. At the same time, energy dependent processes, such as presynaptic reuptake of excitatory amino acids, are disrupted resulting in excitotoxicity by accumulation of glutamate and subsequent Ca^2+^ overload (Kyle and Saha, 2014). Glutamate acting continuously at its NMDA and AMPA receptors evokes Na^+^ and Cl^-^ inward, water flowing along with the ions resulting in cytotoxic edema (Dirnagl et al., 1999). Also, peri-infarct depolarizations can spread energy imbalance through brain cells (Kunz et al., 2010).

The increase in intracellular Ca^2+^ concentration initiates a series of cytoplasmic and nuclear events that result in cellular damage, such as the production of ROS. ROS accumulation, particularly after reperfusion, will cause mitochondrial (Da Silva-Candal et al., 2017) and cellular membrane damage (Dugan and Choi, 1994). Intracellular signaling pathways triggered during excitotoxicity also stimulate the production of pro-inflammatory mediators (Dirnagl et al., 1999). All of these events happening together, after ischemia and also during reperfusion, will contribute to the progression of tissue damage and culminate with cellular death by either necrosis or apoptosis (Kunz et al., 2010).

The changes described above do not homogenously affect the ischemic territory. In the lesion core, where there is a reduction of approximately 80% of blood flow (Hossmann, 1994), permanent damage happens minutes after the ischemic event and cells are rapidly killed. In between this region and the normal tissue is the penumbra where there is still salvageable tissue, being thus the region of interest when it comes to neuroprotection (Dirnagl et al., 1999).

The events described so far, such as excitotoxicity, oxidative stress and inflammation, also affect the BBB structure. The BBB plays a vital role in regulating the traffic of fluid, solutes and cells at the blood-brain interface and maintaining the microenvironment homeostasis (Jiang et al., 2017). BBB is composed of brain endothelial cells (BEC) whose main particularity, when compared to other endothelial cells found in the organism, is the existence of an increased number of tight junctions connecting adjacent cells, resulting in a decrease of paracellular transport (Abbott et al., 2006). Thus, BEC form a barrier that protects the brain against possible toxic substances (Azad et al., 2015). Ischemia/reperfusion damage will act on BEC causing structural disruption of tight junctions and contributing to BBB increased permeability and dysfunction (Jiang et al., 2017).

Therefore, following an ischemic stroke, blood-borne cells, chemicals and fluid extravasate into brain parenchyma (Keaney and Campbell, 2015). The water and ion homeostasis of the brain is also disrupted, leading to cerebral vasogenic edema (Rosenberg, 1999). Infiltrating leukocytes exacerbate inflammatory responses and aggravate brain injury (Huang et al., 2006). Besides all the detrimental consequences of BBB disruption, one potential benefit is that it may facilitate therapeutic agents to reach the brain (Jiang et al., 2017).

## Current Limitations in the Diagnosis and Therapy of Ischemic Stroke

The reduced permeability of BBB to most drugs, including imaging contrast agents, macromolecular compounds, nucleic acids and proteins (Hawkins and Davis, 2005; Gabathuler, 2010; Essig et al., 2012; Tam et al., 2016) represents a major obstacle to the development of safe and effective diagnostic and treatment strategies for ischemic stroke (Mouhieddine et al., 2015). Even knowing that during ischemia/reperfusion there is a rupture in the BBB structure, this opening is transitory and the amount of drug that can actually reach the tissue is often not enough to produce the desired effect, especially in the case of macromolecular drugs (Chen and Gao, 2017; Merali et al., 2017). In this context, there has been much interest in the design of nanoparticles capable of carrying therapeutic and diagnostic agents across the injured and normal BBB and targeting the ischemic tissue and the penumbra region of the damaged tissue (Kafa et al., 2015; Mendonça et al., 2015; Fukuta et al., 2016; Zhao Y. et al., 2016).

Essentially two neuroimaging techniques are routinely used for the diagnosis and surveillance of patients suspected of acute ischemic stroke: X-ray computed tomography (CT) and multimodal magnetic resonance imaging (MRI) (Wang et al., 2016).

Although CT is widely available and less expensive, it is not sensitive for detecting ischemic stroke and distinguishing new events. Indeed, less than a third of patients with brain ischemia exhibits characteristics from CT findings within 3 h of symptoms onset (Chalela et al., 2007). Moreover, the fact that stroke-like symptoms may also be present in a wide range of other non-vascular diseases like epileptic seizures and migraine, makes difficult the accurate diagnosis and decision for administration of a thrombolytic drug (Mouhieddine et al., 2015).

Conventional MRI is a vital and versatile imaging tool in clinical practice, offering advantages for the assessment of acute stroke, especially with diffusion-weighted imaging (DWI) (Chalela et al., 2007; Latchaw et al., 2009; Merino and Warach, 2010). However, the efficiency of detection of an ischemic stroke within 3 h of symptoms onset is around 70% (Chalela et al., 2007).

Although a more accurate diagnostic of ischemic stroke can still be achieved through the combined use of CT and MRI (Brazzelli et al., 2009), there is a great need for a more sensitive technique. In this scenario, much effort has been devoted to the search for serum biomarkers, new imaging methods and the improvement of contrast agents through nanotechnology (Mouhieddine et al., 2015; Wang et al., 2016).

Regarding therapeutic options for acute ischemic stroke, only two are available: thrombolysis and mechanical thrombectomy, both strategies focusing on reperfusion therapy. Thrombolysis is the leading treatment for acute stroke, performed by a pharmacological intervention using recombinant tissue plasminogen activator or t-PA, the only available treatment approved by the US FDA (Chen and Gao, 2017). However, a small number of stroke victims can actually use t-PA treatment since it has a narrow time window of treatment efficacy on the eligible patients (4–5 h of symptom onset), and an increased risk of intracerebral hemorrhage (Khandelwal et al., 2016).

Mechanical thrombectomy utilizes a surgical process to mechanically restore the blood flow in large cerebral arteries promoting a more effective recanalization than thrombolysis (Chamorro et al., 2016). Although the mechanical procedure has shown some advantages when compare to t-PA treatment alone (Albers et al., 2018) it is not available in every hospital, reducing the number of patients who can receive endovascular treatment with mechanical thrombectomy (Chamorro et al., 2016).

Moreover, even when recanalization is successful in installing reperfusion, there is still left sequels that can impair patients life quality, as reperfusion itself can induce ROS production and tissue damage (Manzanero et al., 2013). Aiming to protect brain tissue against ischemia/reperfusion damages, neuroprotective strategies have been studied to extend neurons survival, increase the therapeutic window, and induce neurological repair improving functional outcomes (George and Steinberg, 2015).

Several promising neuroprotective drug candidates have been identified in rodent models. However, most of the strategies established in animal models has failed in clinical trials (George and Steinberg, 2015). The poor methodological rigor in some preclinical studies and the inappropriate use of animal models when simulating the patient conditions might have contributed to the failure in translation of rodents results into clinical success (Feng and Belagaje, 2013).

Moreover, the complexity of the physiopathological events that take place during cerebral ischemia and the still limited knowledge about the molecular and cellular mechanisms involved and their spatial and temporal occurrence represent an obstacle to the identification of effective tissue biomarkers and the design of powerful nanotechnology-based strategies for diagnosis and therapy (Da Silva-Candal et al., 2017).

In summary, the diagnosis and therapy of ischemic stroke need to be improved and, in that sense, a critical step seems to be the safe and effective delivery of substances to the ischemic region (Mouhieddine et al., 2015). In this context, the design of tailored nanoparticles that could ameliorate the drug delivery and the existing imaging techniques with higher sensibility, specificity and spatial and temporal resolution could bring great benefits to stroke diagnosis and treatment. Along with that, the development of reversible, biocompatible and direct nanobiosensors for long term use in animal and tissue models of brain ischemia would be of great interest to provide knowledge about the pathophysiology of stroke and contribute to the development of more specific and effective treatments.

**FIGURE 1:**
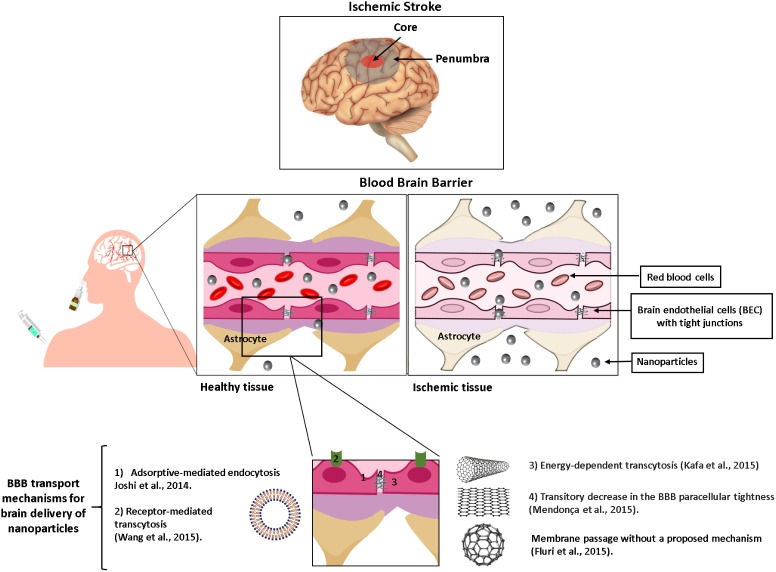
Schematic representation of the brain tissue and the strategies used with liposomes or carbon nanomaterials to improve drug delivery into the ischemic regions (core and penumbra) following intravenous or intranasal route. The BBB is shown before and after an ischemic event.

As illustrated in **Figure 1** and discussed in details below, liposomes and carbon nanomaterials have been extensively studied as nanomaterials for application against ischemic stroke and several different strategies have emerged with promising results.

## Liposomes as Drug Nanocarriers

Liposomes are the first generation of drug nanocarriers (Budai and Szógyi, 2001). They are vesicles composed of concentric lipid bilayers, which are separated by water compartments. They are constituted of natural or synthetic lipids with amphiphilic nature: a polar head group covalently attached to one or two hydrophobic hydrocarbon tails (Frézard, 1999). These lipids are usually biocompatible and biodegradable and resemble those found in biological membranes (Masserini, 2013; Lamichhane et al., 2018). Liposomes can be found in the composition of several pharmaceutical products already marketed for treatment of cancer, fungal infections, and as immunoadjuvant in vaccines (van Hoogevest and Wendel, 2014).

Liposomes are classified according to their size and number of lamellae: small unilamellar vesicles (SUV) with a size up to 100 nm and one bilayer; large unilamellar vesicles (LUV) with a size of more than 100 nm and one bilayer; and multilamellar vesicles (MLV) that can reach several μm and are made of multiple concentric bilayers (Masserini, 2013).

**FIGURE 2:**
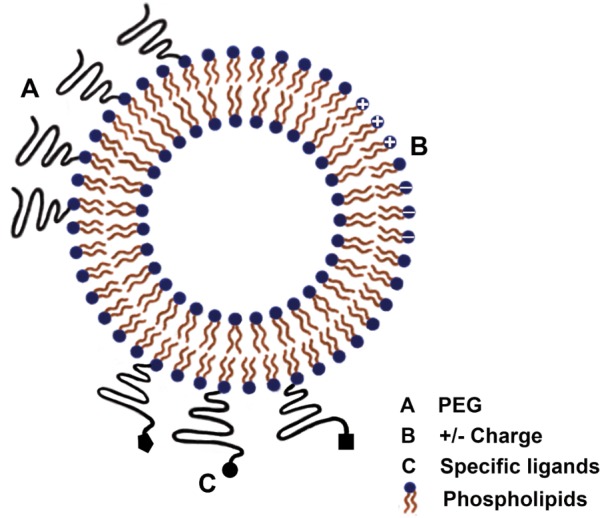
Schematic representation of different liposome membrane functionalization strategies for application against ischemic stroke: PEGylation; cationic and/or anionic charges; and specific targeting ligands for BEC or ischemic tissue receptors.

As illustrated in **Figure 2**, liposomes are highly versatile, allowing accommodation of hydrophilic drugs in the aqueous space or hydrophobic drugs in the lipid bilayer and surface modifications to control their interaction with biological environments and fate (Li et al., 2017). Once the drug is inside the liposome, it is protected against physiologically occurring events, such as enzymatic degradation, immunological and chemical inactivation and fast plasma clearance, resulting in enhancement and prolongation of its action. Another common benefit of liposome encapsulation is the decreased drug exposure of healthy tissues, reducing the side effects of the treatment (Bozzuto and Molinari, 2015). Thus, because of all of the characteristics discussed above, liposomes represent the most well-studied system for drug delivery to treat CNS disorders (Vieira and Gamarra, 2016).

Liposomes have been widely studied for use in stroke therapy, as shown in **Table 1**. Imaizumi et al. (1990) performed one of the precursor studies with liposomes in an animal model of focal ischemic stroke. Once superoxide dismutase (SOD), a free radical scavenger, has a short half-life and is unable to cross the BBB, this enzyme was administered in the liposome-encapsulated form via jugular vein in rats submitted to middle cerebral artery occlusion (MCAO). Brains of animals injected with the liposome formulation showed increased levels of SOD and a reduced infarct volume (Imaizumi et al., 1990).

**Table 1:** Neuroprotective agents tested under liposomal form against ischemic stroke.

Neuroprotective agent	Liposome characteristics	Stroke model	Main findings	Reference
Panax notoginsenoside	Core-shell hybrid liposomal vesicles	Bilateral common carotid artery occlusion (BBCAO)	Oral administration with inhibition of brain edema, reduction of infarct volume and increase in superoxide dismutase	Zhang et al., 2012
t-PA/ dexamethasone	PEGylated liposomes	MCAO by thromboembolic ischemia	Intravenous injection with improvement of behavioral outcome	Tiebosch et al., 2012
FK506	PEGylated liposomes	Transient middle cerebral artery occlusion (t-MCAO)	Intravenous injection with accumulation of liposomes in the brain parenchyma, reduction of cerebral cell death and improvement of motor function deficits	Ishii et al., 2013
Citicoline	Targeted PEGylated immunoliposomes labeled with gadolinium	Permanent intracranial occlusion of middle cerebral artery	Intravenous injection with accumulation of 80% vectorized liposomes in the periphery of the ischemic lesion as detected by MRI and reduction of lesion volumes up to 30% in comparison to animals treated with the free drug	Agulla et al., 2014
Basic fibroblast growth factor (bFGF)	Gelatin-cored liposomes	Transient middle cerebral artery occlusion (t-MCAO)	Intranasal administration with increased accumulation of bFGF in the brain, improved neurological score and reduced infarct volume	Zhao Y.Z. et al., 2016
ZL006	Targeted PEGylated liposomes	Transient middle cerebral artery occlusion (t-MCAO)	Liposomes with targeting peptide ligands for both transferrin receptor and stroke tissue accumulated in the brain, ameliorated infarct volume, neurological deficit and histopathological severity in MCAO injury	Zhao Y. et al., 2016
Fasudil	PEGylated liposomes	Transient middle cerebral artery occlusion (t-MCAO)	Intravenous injection with accumulation of liposomes in the ischemic region, amelioration of ischemic/reperfusion injury and motor score	Fukuta et al., 2016
Simvastatin	PEGylated liposomes	Transitory middle cerebral artery occlusion (t-MCAO)	Intravenous injection of neutral and negatively charged liposomes reaching the brain, accumulating in the infarcted area and delivering simvastatin to the brain.	Campos-Martorell et al., 2016
Citicoline	Targeted PEGylated immunoliposomes	Transient middle cerebral artery occlusion (t-MCAO)	Intra-arterial or intravenous injection of citicoline-liposomes (PEGylated or conjugated to targeting vascular cell adhesion molecule 1), with direct detection through the CEST-MRI in the ischemic areas, being a potential theranostic device	Liu et al., 2016
Cyclosporine	PEGylated liposomes	Transient middle cerebral artery occlusion (t-MCAO)	Intravenous injection of CsA-liposomes showing recovery of the infarct size, brain edema and neurological activities, and inhibition of inflammation	Partoazar et al., 2017
t-PA/fasudil	PEGylated liposomes	MCAO by photochemically induced thrombosis (PIT)	Intravenous administration of fasudil-lip before t-PA decreased the risk of t-PA-derived cerebral hemorrhage and extended the therapeutic time window of t-PA	Fukuta et al., 2017
Hemoglobin	PEGylated liposomes	MCAO by transorbital approach	Intravenous administration to nonhuman primates of liposomal hemoglobin was effective in reducing the area of histological damage in the brain cortex	Kawaguchi et al., 2017
Paired immunoglobulin-like receptor B ectodomain (sPirB)	PEGylated liposomes labeled with NIR probe	Transient middle cerebral artery occlusion (t-MCAO)	Intravenous administration of sPirB-containing liposomes with accumulation in the ischemic region and improved ischemic stroke model recovery, showing potential for a new theranostic platform	Wang et al., 2018

Blood brain barrier is one of the major issues in the treatment of stroke. It is crucial that liposomes can cross the BBB and it is also desirable that they remain in the bloodstream for a long period of time, so as to deliver a sufficient quantity of drug to the brain tissue (Moghimi et al., 2001). The circulation time of liposomes can be enhanced through reduction of particle size or modification of their surface by polyethylene glycol (PEG) (Fukuta et al., 2014; Bozzuto and Molinari, 2015; Wang et al., 2015; Campos-Martorell et al., 2016; Chen and Gao, 2017). It has been reported that PEGylated liposomes can accumulate into ischemic brain regions after a stroke event (Fukuta et al., 2016), presumably because of the disruption of the BBB and the enhanced vesicle permeation and retention at the ischemic site. Fukuta et al. (2014) have shown that PEGylated liposomes detection increased in the ischemic region over time period, even when there was little blood flow in the affected area. In another work, Ishii et al. (2013) evidenced that a single injection of PEGylated liposomes containing the immunosuppressant FK506 at a low dosage in MCAO rats significantly reduced cerebral cell death and ameliorated motor function deficits. Interestingly, liposome encapsulation of FK506 was found to decrease the drug toxicity that has been responsible for the failure of clinical trials of this drug against stroke (Ishii et al., 2013)

The surface charge is another factor that can influence the accumulation of liposomes into the brain. Campos-Martorell et al. (2016) showed that neutral and negatively charged PEGylated liposomes administered intravenously were captured to a lower extent by the liver and lungs in comparison to cationic vesicles. As consequence, the neutral and anionic liposomes showed prolonged circulation time in the blood and higher uptake in the ischemic region (Campos-Martorell et al., 2016). In another study, non-PEGylated liposomes with different surface charges were given through intracarotid injection as an attempt to achieve early brain deposition. The brain accumulation of these liposomes following intra-arterial injection was more effective from cationic vesicles than anionic or neutral ones, possibly due to the electrostatic interactions between the cationic liposomes and negatively charged cell surface, enhancing nanoparticle uptake by adsorptive-mediated endocytosis (Joshi et al., 2014).

An additional option to further increase the transport of drugs across the BBB is the coupling of specific ligands to the liposome surface in order to target surface proteins constitutively expressed at the BBB, such as low-density lipoprotein receptor, glucose transporter (GLUT1), transferrin or insulin receptors (Gabathuler, 2010; Ying et al., 2010; Spuch and Navarro, 2011). This kind of surface modification can increase liposome uptake by the BEC through receptor-mediated transcytosis, resulting in greater amount of drug that reaches the brain (**Figure 1**) (Wang et al., 2015). Following this approach, Zhao Y. et al. (2016) investigated a dual targeting strategy for ischemic stroke treatment, using liposomes containing a neuroprotectant (ZL006) with their surface decorated with a transferrin receptor-derived peptide ligand (T7) to improve the passage across the BBB and a stroke-homing peptide (SHp) to target the ischemic region. T7&SHp-P-L/ZL006 liposomes decreased the infarct volume, neurological deficit, and histopathological severity in the MCAO rat model (Zhao Y. et al., 2016).

Zhang et al. (2018) reported that liposomes with Cyclo(Arg-Gly-Asp-D-Phe-Cys) (cRGD) covalently coupled to the membrane surface can bind to the activated platelets while not to the resting platelets. The authors demonstrated that the cRGD liposomes containing urokinase could improve the thrombolytic efficacy by almost fourfold over free uroquinase, showing potential for treatment of ischemic stroke (Zhang et al., 2018).

Among the neuroprotective drug candidates for ischemic stroke, those that can activate the ACE2-Ang-(1-7)-Mas axis of the Renin Angiotensin System (RAS) are of special interest (Bennion et al., 2015b). The heptapeptide, angiotensin-(1-7) [Ang-(1-7)], final endogenous effector of ACE2-Ang-(1-7)-Mas pathway, has shown neuroprotection in several models of ischemic stroke (Zhang et al., 2008; Mecca et al., 2011; Bennion et al., 2015a, 2018). On the other hand, the rapid *in vivo* metabolism of the peptide through proteolytic inactivation results in a short plasma half-life (less than 1 h) and biological actions, limiting its therapeutic potential. Furthermore, the high molecular weight and hydrophilic character of Ang-(1-7) prevent its absorption across biological barriers, such as BBB. Interestingly, it was shown that encapsulation of Ang-(1-7) into PEGylated liposomes prolonged the peptide biological action from 8 min to 5 days following microinjection into the rostral ventrolateral medulla (RVLM) of normotensive rats (Silva-Barcellos et al., 2004). This data strongly suggests that such liposome formulation of Ang-(1-7) may find application in the treatment of ischemic stroke.

The intranasal route has recently gained, attention once it is a region that has free access to the brain tissue by the olfactory and trigeminal nerve and it also constitutes a non-invasive alternative route (Zhao Y.Z. et al., 2016). Besides that, intranasal drug delivery provides decreased systemic exposure of the drug and limited degradation of therapeutics (Meredith et al., 2015). In this context, the use of nanoparticles also brings significant benefits, such as increase of mucoadhesion providing sustained and controlled drug release, enhanced drug deposition at olfactory epithelium and improved nasal drug absorption (Illum, 2000; van Woensel et al., 2013). In addition of promoting the accumulation of drug into the brain, liposomes can decrease the mucosal drug toxicity that may be unleashed when intranasal administration is done chronically (Mainardes et al., 2006). It is also noteworthy that cationic liposomes were particularly effective in prolonging the residence time at the nasal cavity and assisting transport of proteins across nasal mucosa (Zheng et al., 2015). Furthermore, PEGylated liposomes enhanced the drug bioavailability and showed greater residence time when compared to the conventional non-PEGylated liposomes (Khan et al., 2017). As another strategy, Zhao Y.Z. et al. (2016) prepared gelatin-cored liposomes encapsulating basic fibroblast growth factor (bFGF), a potential protective substance for patients with stroke. The bFGF-liposomes applied intranasally in a rat model of cerebral ischemia/reperfusion improved bFGF accumulation in brain tissues and promoted functional recovery of the animals (Zhao Y.Z. et al., 2016).

Nanotheranostics is the field that combines at the same time nanoparticle-mediated therapy and the study of nanoparticles localization and fate usually through imaging (Mouhieddine et al., 2015). Agulla et al. (2014) have identified the heat shock protein-72 (HSP72) as a suitable biomarker for the peri-infarct region and described the development of anti-HSP72 stealth immunoliposomes labeled with gadolinium and containing the neuroprotectant citicoline. The viability of this nano-platform for the diagnostic by MRI and therapy of cerebral ischemia was established in an animal model (Agulla et al., 2014). In a more recent study, Liu et al. (2016) proposed citicoline-liposomes as a prototype theranostic system, after evidencing that their accumulation can be detected in the ischemic brain using Chemical Exchange Saturation Transfer (CEST)-MRI. Citicoline is a precursor of phosphatidylcholine that has a cytosine in its structure, which permits its detection by CEST-MRI. The more abundant expression of vascular cell adhesion molecule 1 (VCAM-1) on inflamed vessels in the ischemic brain led these authors to further design anti-VCAM-1 immunoliposomes that promoted greater accumulation of citicoline in the brain of ischemic rats, in comparison to non-targeted liposomes. However, the uptake of these targeted liposomes in the rat brain after ischemic injury was approximately fourfold lower and showed a more dispersed distribution after intravenous administration, when compared to intra-arterial injection.

After observing the increased expression of paired immunoglobulin-like receptor B (PirB) in the ischemic hemisphere of mice 24 h post-MCAO, Wang et al. (2018) constructed anti-PirB immunoliposome probe with a near-infrared fluorophore that was successfully applied to *in vivo* imaging for upregulated PirB region in a cerebral ischemic stroke model. These authors also used soluble PirB ectodomain, as a therapeutic reagent encapsulated in liposomes, showing a significant functional recovery in a model of ischemic stroke.

Wen et al. (2012) have investigated PEGylated liposomes for brain drug targeting and imaging, after encapsulation of apomorphine in the internal aqueous compartment and quantum dot (QD) in the liposomal membrane. As main advantages over conventional organic fluorophores, semi-conductor-based QDs have narrow band emissions together with large ultraviolet absorption spectra, which enable multiplex imaging under a single light source. The QD- and drug-containing liposomes were found to accumulate into the brain of mice, as evidenced by QD fluorescence imaging and drug quantification in the tissue, showing potential for imaging and treating brain disorders (Wen et al., 2012).

Several concerns will need to be overcome before engineered liposomal nanocarriers can be incorporated into everyday clinical practice. The ideal nanoparticles for use as contrast agents must be detectable at reduced doses, should cross the BBB and target a time-accurate biomarker. It must also be non-toxic and preserve cellular functions. Hence, contrast agents associated to liposomes should be carefully chosen, or eventually optimized, so as to avoid adverse effects. For instance, a recent work emphasized the potential toxicities of QDs due to their specific metallic composition, the risk of release of toxic metal ions after prolonged exposure of biological systems and several undefined factors of nanoparticles themselves (Wang and Tang, 2018). Thus, much effort is still needed regarding the achievement of biocompatible QDs through surface modification and the understanding of their mechanisms of toxicity, before they can be considered for use in humans. Considering that MRI is routinely used in the clinic for diagnosis of stroke, the translational potential of liposome-based MRI contrast agents seems currently greater than that of fluorescence-based imaging agents.

It is also important to take into account that once the nanocarrier has reached its target, the encapsulated drug still has to be released to exert its pharmacological action. Three distinct mechanisms can contribute to the *in vivo* release of the encapsulated drug from liposomes: (i) the spontaneous simple diffusion of the drug across the liposome bilayer; (ii) the endocytosis of liposomes by cells, their degradation by lysosomal phospholipases and the subsequent release of the drug in the cytosol or the extracellular medium after exocytosis; (iii) the induction of drug release from liposomes by specific stimuli, such as external magnetic field or focused ultra-sound, variations in temperature or pH, depending on the characteristics of liposomes (Vieira and Gamarra, 2016). Since there is a decrease in the pH at the ischemic region (pH<6.75) (Anderson et al., 1999), it would be interesting to explore pH-sensitive liposomes to improve the release of neuroprotective agents or specific image markers into the ischemic region. In agreement with this proposal, pH-sensitive liposomes have already shown good results in the delivery of anticancer drugs in the more acidic environment of tumors (Ferreira et al., 2013). Such liposomes were first prepared from the mixture of dioleoylphosphatidylethanolamine, a lipid with a carboxylic acid head group and a PEGylated lipid (Simões et al., 2004). More recently, Pacheco-Torres et al. (2015) incorporated an engineered ion channel in the liposome membrane, which could discriminate physiologically relevant minor pH changes with an unprecedented precision of 0.2 pH unit, and release the drug accordingly in the tumor site.

## Carbon-Based Nanomaterials

### General Physicochemical Characteristics

**FIGURE 3:**
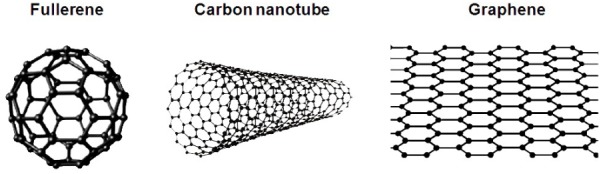
Schematic representation of carbon-based nanomaterials that, due to their unique properties, have shown potential applications in the treatment and diagnosis of stroke: fullerene (hollow sphere of carbon atoms in sp^2^ hybridization); carbon nanotube (tubular structure of carbon atoms in sp^2^ hybridization); and graphene (single layer of carbon atoms arranged in a hexagonal lattice in sp^2^ hybridization). Reprinted (adapted) with permission from Hong et al. (2015).

Carbon nanomaterials (CNMs) have been the subject of intense research during the last 30 years due to their unique properties related to the quantum confinement of the electrons movement at discrete energy levels in the nanometric structure. The most studied allotropes are the sp^2^ hybridized carbons (**Figure 3**): fullerenes (zero-dimensional, 0D), carbon nanotubes (CNTs, one-dimensional, 1D) and graphene (two-dimensional, 2D), although structures such as carbon dots and nanodiamond have been reported (Xu et al., 2004; Mochalin et al., 2012). Fullerenes (C60) were first described when Kroto et al. (1985) reported the production of a stable cluster consisting of 60 carbon atoms by vaporization of graphite using laser irradiation.

Carbon nanotubes, tubular carbon structures with nanometric diameter, gained attention of the scientific community with the publication of Iijima (1991) reporting the synthesis of “helical microtubules of graphitic carbon” by arc-discharge evaporation method. Graphene, a two-dimensional single layer of carbon atoms, was reported by Novoselov et al. (2004), who described the preparation of monocrystalline graphitic films of few atoms thickness (including single layer) by mechanical exfoliation of highly oriented pyrolytic graphite. Nowadays these nanomaterials are widely employed in different fields including among others: communication, energy, military, aerospace, and nanomedicine (De Volder et al., 2013). However, for practical applications it is imperative to modify the surface of these materials to allow integration with the desired medium.

**FIGURE 4:**
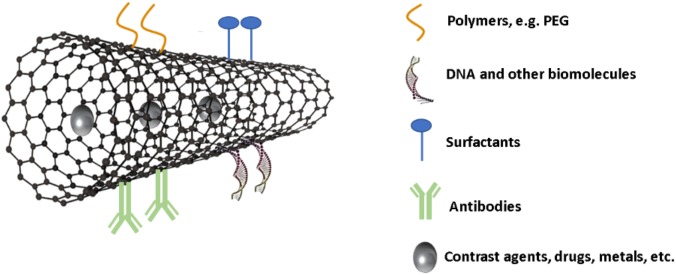
Schematic representation of surface functionalization and loading of carbon nanotubes for biomedical applications.

For biological applications, surface modifications (covalent or non-covalent) allow better dispersion of the nanomaterial in physiological medium and ensure biocompatibility. In a wider perspective, this approach can confer functional characteristics to the CNMs. The conjugation of the nanomaterials with different molecules, such as polymers, proteins, different DNA sequences or other specific compounds, can generate different functions for *in vivo* applications, such as sensors, biomarkers, drug carrier for therapeutics (**Figure 4**). The purification of the CNMs is an earlier step that is crucial for elimination of residues or by-products, such as metal particles of catalysts used in the synthetic procedure amorphous carbons and other impurities that may exert toxicity.

### Potential Therapeutic Applications

Some of the main advantages of CNTs, when compared to other drug nanocarriers, are their high specific area, allowing binding of multiple copies of different molecules onto their surface, and their propensity to cross different biological barriers, entering the cytoplasm either passively through ‘nanoneedle’ mechanism or through endocytosis, which allows the transport and delivery of drugs and macromolecules (Tîlmaciu and Morris, 2015). The mechanism of uptake of CNTs appears to vary with functionalization, length, diameter, number of walls, and concentration of CNTs. Indeed, the unique ‘nanoneedle’ transport mechanism has been reported for certain types of single-walled carbon nanotubes (SWCNTs), whereas multi-walled carbon nanotubes (MWCNTs) are more likely to be endocytosed because of their larger diameter (Kafa et al., 2015; Mehra and Palakurthi, 2016). The intrinsic spectroscopic properties of CNTs, such as Raman and photoluminescence, afford additional advantages for real-time monitoring of drug delivery efficacy *in vitro* and *in vivo* (Lamprecht et al., 2012).

After SWCNTs given orally in a rodent Alzheimer’s disease model were found to successfully deliver acetylcholine into the brain (Yang et al., 2010), CNTs started to be considered as potential tools in the treatment of CNS diseases. Other works have established the direct ability of CNTs to cross the BBB. Using an *in vitro* co-culture BBB model (primary porcine brain endothelial cells, PBEC), Kafa et al. (2015) brought evidence of the permeation of functionalized multi-walled carbon nanotubes (f-MWCNTs), more specifically MWCNT-NH_3_^+^, across the cell monolayer via energy-dependent transcytosis. In another study, f-MWCNTs were shown to enter into the brain via endothelium, following systemic injection in rodents, with accumulation not just in endothelium but also in brain parenchyma (Costa et al., 2016).

Carbon nanotubes can be used either for their own action on the nervous tissue or their ability to carry other drugs (**Table 2**). Lee et al. (2011) reported for the first time the neuroprotective effects of SWCNTs (without any therapeutic molecule), by showing that pretreatment of rats with MCAO ischemic brain injury through intra-ventricular administration of amine-modified single-walled carbon nanotubes (a-SWCNTs) could protect neurons and enhance the recovery of behavioral functions. Evidence was obtained that the Akt pathway and the maintenance of cell-to-cell interactions (higher levels of N-Cadherin) contributed to the protective action of a-SWNTs (Lee et al., 2011).

**Table 2:** Carbon based nanomaterials tested in ischemic stroke models.

Carbon nanomaterial	Functionalized derivative	Stroke model	Main findings	Reference
Single walled-carbon nanotubes (SWCNT)	Amine-modified SWCNT	Transient middle cerebral artery occlusion (t-MCAO)	Pretreatment with a-SWCNT (lateral ventricle injection) protected animals following ischemia/reperfusion	Lee et al., 2011
Multi walled-carbon nanotubes (MWCNT)	Hydrophobic MWCNT impregnated with subventricular zone neural progenitor cells (SVZ NPCs)	Transient middle cerebral artery occlusion (t-MCAO)	HP CNT-SVZ NPC transplants (microinjection into striatum post ischemia) improved rat behavior and reduced infarct cyst volume and infarct cyst area	Moon et al., 2012
Fullerene	Hexasulfobutylated C_60_ (FC_4_S)	Unilateral middle cerebral artery occlusion	Intravenous administration of FC4S reduced the total volume of infarction in both pretreatment (15 min before MCAO) and treatment (injected when the common carotid arteries clips were removed) groups	Huang et al., 2001
Fullerene	Carboxyfullerene	Transient middle cerebral artery occlusion (t-MCAO)	Intracerebroventricular infusion of carboxyfullerene attenuated oxidative injuries and cortical infarction. No protection of cortical infarction was observed after intravenous administration of carboxyfullerene. Undesired effects need to be considered	Lin et al., 2002
Fullerene	Polyhydroxylated fullerene	Transient middle cerebral artery occlusion (t-MCAO)	Administration of fullerene nanoparticles before and after MCAO significantly decreased the infarct volume and inhibited brain oxidative/nitrosative damage.	Vani et al., 2016
Fullerene	Fullerenols (OH-F) and glucosamine fullerenes (GlcN-F)	Transient middle cerebral artery occlusion (t-MCAO)	Intravenous injection of OH-F and GlcN-F prevented neuronal loss in the perilesional area and lead to a reduction in inflammation after stroke	Fluri et al., 2015

In addition to the neuroprotective action of the nanotube itself in a model of ischemia, this nanomaterial was also effective when impregnated with progenitor cells. An *in vivo* study has shown that hydrophobic carbon nanotubes (HPCNT) impregnated with subventricular zone neural progenitor cells (SVZ NPCs) could repair damaged neural tissue following stroke. HPCNT-SVZ NPCs transplanted rats exhibited improved behavior and reduced volume and area of infarct cyst, compared to experimental control. The majority of the transplanted HPCNT-SVZ NPCs collectively broadened around the ischemic injured region and the SVZ NPCs differentiated into mature neurons, attained the synapse morphology (TUJ1, synaptophysin), and decreased microglial activation (CD11b/c [OX-42]). This study pioneered the concept that CNTs can improve stem cell differentiation, leading to heal stroke damage (Moon et al., 2012).

Al-Jamal et al. (2011) have confirmed that CNTs have also a potential to deliver siRNA into the brain, through demonstration of functional recovery in endothelin-1 stroke murine model after stereotaxic administration of siRNA (for caspase 3 silencing) complexed with amino-functionalized MWNT.

Fullerene derivatives were repeatedly reported as neuroprotective in *in vitro* and *in vivo* stroke models (Huang et al., 2001; Lin et al., 2002; Fluri et al., 2015; Vani et al., 2016). Hexasulfobutylated fullerene (C60 FC4S), when injected intravenously before and during MCAO in Long-Evans rats, promoted the increase of nitric oxide content and the decrease of LDH levels and total volume of infarction, presumably through action as free radical scavenger (Huang et al., 2001). Intracerebroventricular infusion of carboxyfullerene in rats submitted to MCAO stroke attenuated cortical infarction and prevented the elevation of lipid peroxidation and depletion of GSH level induced by transient ischemia/reperfusion. However, adverse effects and death were observed in some cases (Lin et al., 2002). In accordance with these works, Vani et al. (2016) showed that polyhydroxylated fullerene or fullerenol (OH-F) derivatives protected rat brain cells against ischemia/reperfusion injury and inhibited brain oxidative/nitrosative damage in a MCAO model, acting as a potent scavenger of free radicals. Also, Fluri et al. (2015) reported that fullerenol and glucosamine-fullerene conjugate (GlcN-F) led to a reduction of cellular damage and inflammation after stroke. In this case, fullerenol worked as a radical scavenger and the glucosamine derivative reduced inflammation (Fluri et al., 2015).

Another recently discovered nanomaterial, graphene, exhibited unique biological actions, with potential application in stroke treatment. In the study conducted by Mendonça et al. (2015) reduced graphene oxide (rGO) was found to reach the thalamus and hippocampus of rats following systemic injection. The entry of rGO involved a transitory decrease in the BBB paracellular tightness, as evidenced by extravasation of vital Evan’s Blue stain into the brain (Mendonça et al., 2015). Importantly, the rGO-induced transitory opening of the BBB seems not to cause major deleterious effects. Although stroke leads to a disruption of the BBB, we cannot control the duration and extent of this phenomenon. The temporary permeabilization of the BBB caused by rGO may allow intentional enhancement of brain uptake of delivery systems for diagnostic or therapeutic purposes. Thus, rGO may be used to allow a controlled therapeutic window for carrying drugs into the ischemic site.

In order to fully address the potential of CNTs for the treatment of brain ischemia, one should also consider their toxicity, its possible causes and ways to overcome it. When used in their pristine state, directly after synthesis, CNTs contain impurities, aggregate into bundles in aqueous media and strongly interact with biomolecules, leading to severe toxic effects. Nonetheless, when purified and surface-functionalized, their toxicity is drastically decreased. It is now well established that characteristics of CNTs such as degree and type of functionalization, purity, shape, stability, surface reactivity and agglomeration state exert a marked influence on their biological and toxic effects (Grabinski et al., 2007; Johnston et al., 2010; Zhang et al., 2010, 2011). Several studies also support the model that the main mechanism of toxicity of these nanomaterials is the induction of oxidative stress (Murray et al., 2009; Pichardo et al., 2012; Shvedova et al., 2012; Liu et al., 2014; Weber et al., 2014). The use of dense functionalization, biocompatible polymers and highly purified materials generating aqueous-stable dispersions was found effective to minimize this toxicity (Liu et al., 2008; Zhang et al., 2011; Iverson et al., 2013; Mendonça et al., 2015; Calle et al., 2018).

### Biological Imaging and Nanobiosensors

There is still a great need of methods to create a precise, accurate and space-time resolution detection for investigating the changes in the brain tissue after an ischemic event. The rapid development of nanotechnology has led to promising diagnostic tools for stroke using engineered nanomaterials. In addition to the diagnosis of patients, this technology may aid in understanding the biochemical and pathophysiological mechanisms of stroke using *in vitro* and *in vivo* model.

Honjie Dai’s group developed a new method to image mouse cerebral vasculature without craniotomy, using through-scalp and through-skull fluorescence imaging in a biological transparent sub-window in the 1.3–1.4 μm wavelength range (the NIR-IIa region). They exploited the intrinsic photoluminescence of single-walled carbon nanotubes (SWNT–IRDye800). This technique allows a fluorescence imaging to a depth of >2 mm, sub-10-μm resolution and imaging rate of ∼5.3 frames per second providing a non-invasive, real-time assessment of blood flow anomaly with high spatial and temporal resolution in a MCAO stroke model (Hong et al., 2014).

Besides the development of new tools for the diagnosis of patients, another equally important approach is the use of biosensors to uncover the biochemical and pathophysiological mechanisms of stroke in animal and cells models. In this scenario, optical CNT-based biosensors show great promise, because of their unique fluorescence properties, allowing high spatio-temporal resolution at biologically relevant concentrations (Soleymani, 2015; Polo and Kruss, 2016).

Strano’s group has studied and developed a new class of biosensors: fluorescent sensors for the detection of biomolecules, formed from single-wall semiconductor carbon nanotubes conjugated to polymers, called CoPhMoRe (corona phase molecular recognition) (Kruss et al., 2013; Zhang et al., 2013). These SWCNT-based biosensors operate basically with the fluorescence quenching and/or peak shift in the NIR-II window, as a response to surface adsorption events of certain molecules. As discussed above, the SWCNT fluorescence in the near infrared (nIR) is ideal for biomedical applications because it is in the window of optical transparency of biological tissues (O’connell et al., 2002). The most used polymers attached to the sensor are specific DNA sequences. As demonstrated in the work of Kruss et al. (2014), DNA oligonucleotides bound to PEG macromolecules (to enhance biocompatibility) interact with SWCNT by wrapping the oligonucleotide chain to the carbonic surface, producing a biocompatible hybrid that functions as a strong and selective dopamine sensor. In this work, an increase in the sensor intrinsic fluorescence response was demonstrated when in contact with dopamine. Thus, a detection specificity to certain molecular species, such as adenosine 5′-triphosphate (ATP) (Kim et al., 2010), hydrogen peroxide (H_2_O_2_) (Jin et al., 2010), neurotransmitters (Kruss et al., 2014, 2017) and nitric oxide (NO) was achieved (Iverson et al., 2013; Ulissi et al., 2014).

These studies have addressed the detection of these biomolecules *in vitro*, through direct interaction in cell culture. However, as demonstrated by Iverson et al. (2013), it is also possible to apply CoPhMoRe technology *in vivo*. These authors used SWCNTs wrapped with DNA oligonucleotides functionalized with PEG (to enable systemic injections) as an *in vivo* nitric oxide sensor. They used a rodent model injected with RcsX tumor cells to cause liver inflammation and *in situ* generation of NO molecules in the liver. A strong fluorescence quenching effect in the presence of NO free radicals was detected (1 μM detection limit). The tissue autofluorescence and background signals were successfully removed from the characteristic NIR-II signals of the SWCNT reporters, based on their spectral differences. It is also noteworthy that SWCNT-fluorescence exhibits no blinking, no bleaching and a large Stokes shift (Kruss et al., 2013). These promising results support the potential of this technology for investigating the pathophysiological mechanisms of stroke, considering its ability to detect molecules possibly involved in an ischemic event, such as neurotransmitters, NO and H_2_O_2_. The detection at a single-molecule level, long-term sensing and regeneration/reversibility of the sensor response may find applications in new cellular assays for disease diagnostics, molecular signaling, and detection of inflammatory signals.

## Concluding Remarks

Stroke, being one of the leading causes of death and disabilities worldwide, still lacks better ways to diagnose and treat the affected patients. Many studies have focused on neuroprotective drugs to provide guard to the damaged brain tissue and they were successful in improving animals’ behavior and neurological score but translational approaches did not show the same efficiency yet. As discussed here, the techniques currently available for the diagnosis of stroke also lack sensitivity.

In this context, nanotechnology has emerged, with effective means of improving drug delivery to the brain and, more specifically, to the ischemic region. As shown in the present review, there is a great potential of liposomes and carbon nanomaterials for applications in the diagnosis and therapy of ischemic stroke. Interestingly, both types of nanostructures exhibit very distinct characteristics and ranges of applications.

The major advantages of liposomes are their reduced toxicity, excellent biocompatibility and ability to accommodate a large variety of bioactive and contrast agents, with very different physicochemical characteristics. Therefore, several neuroprotective agents have been evaluated under the encapsulated form in liposomes, in experimental models of ischemic stroke. The most promising results, regarding drug delivery to the brain and neuroprotection, were achieved using PEGylated liposomes bearing targeting ligands for BECs and/or ischemic tissues receptors. The proof of concept of nanotheranostic approaches using MRI, fluorescent QD or NIR probes was also established. However, in order to translate experimental results into successful clinical applications, there are still some important issues to be addressed. Regarding the route of administration, intra-arterial route has shown higher drug delivery efficiency to the brain, in comparison to the intravenous or intranasal routes. Thus, it is felt that more effective targeting strategies are still needed to improve therapeutic efficacy of these nanosystems by intravenous or intranasal routes. Another complementary approach would be to select the most effective and least toxic neuroprotectant, like for instance the Ang-(1-7) peptide hormone. Finally, the high complexity of liposome nanosystems and related stability and cost issues make necessary the development of specific nanoplatforms for industrial production. In that sense, the experience acquired in the development of existing pharmaceutical liposome-based product should be useful.

We have shown here that carbon based nanomaterials, including nanotubes, fullerenes and graphene, display great versatility regarding size, morphology and surface physicochemical properties. CNTs started to be investigated for ischemic stroke and potential applications for therapy, biosensing and imaging have been identified based on their antioxidant action, their intrinsic photoluminescence, their ability to cross the BBB, transitorily decrease the BBB paracellular tightness, carry oligonucleotides and cells and induce cell differentiation. Therefore, their unique physicochemical properties and the improvement of functionalization methods, now offer a wide range of potential applications. However, considering that CNTs are not naturally encountered in the human body, an important step for clinical translation is the full evaluation of biodistribution and toxicological effects of these materials (considering shape, size, functionalization, and possible catalytic particles contaminants) after systemic administration. Thus, CNTs are at an early stage of development and much work is still needed to reach practical applications. It is felt that CNTs have their greatest potential as nanobiosensor for investigating the biochemical and pathophysiological mechanisms of stroke using *in vitro* and *in vivo* model, thus contributing to the development of more effective therapies for this pathology.

## Author Contributions

FF and AM defined the outline of the manuscript. AM, GB, and LF wrote the first draft of the Section “Introduction.” AM and LF wrote the first draft of the Sections “Physiopathology of Ischemic Stroke” and “Current Limitations in the Diagnosis and Therapy of Ischemic Stroke.” LF and FF wrote the first draft of the Section “Liposomes as Drug Nanocarriers.” GB wrote the first draft of the Section “Carbon-Based Nanomaterials.” FF and AM fully revised the first draft of the manuscript. All the authors contributed to Abstract, Concluding remarks, and the final form of the manuscript.

## Conflict of Interest Statement

The authors declare that the research was conducted in the absence of any commercial or financial relationships that could be construed as a potential conflict of interest.
